# Sanitary Condition of Public Schools

**Published:** 1892-01

**Authors:** 


### SANITARY CONDITION OF PUBLIC SCHOOLS.

The Medical Society of the County of New York, at its regular meeting, held November 23d, adopted the following resolution, offered by Dr. A. Jacobi : *Resolved*—That the Board of Health be requested to use both its actual authority and its moral influence to secure for the school children a sufficient amount of air, light, and accommodation, and to protect them from dangers to their health unavoidable in the present condition of the public schools.

If some action of this nature should be taken by the medical societies of this city, it might possibly result in stimulating the authorities to proper action. It is a notorious fact that in Buffalo there is an over-crowding in our public schools, as well as an improper ventilation in a number of school buildings, and not a few are inadequately served with water. The three conditions that should be insisted upon by the health authorities with reference to each and every school building within its jurisdiction of this city, should be,—first, an ample supply of fresh air in and about the public schools ; second, proper heating and ventilation of every school room; third, adequate water supply to meet all the necessities and exigencies of the services.

This will be another problem for the new Health Commissioner to solve, and we presume he will address himself promptly to the work upon his induction into office. No more important service can be rendered the city than to put its public schools upon a proper sanitary basis, for education obtained at the expense of health is worse than useless.

